# Supplementary material for: Integrating Clinical Parameters into Thyroid Nodule Malignancy Risk: A Retrospective Evaluation Based on ACR TI-RADS
Source: J Clin Med. 2025 Jul 29;14(15):5352. doi: 10.3390/jcm14155352 (PMC12347102; doi:10.3390/jcm14155352)
Supplement: Supplementary file 1 [file jcm-14-05352-s001.zip › jcm-3743057-supplementary.pdf]

Table S1. Age stratification and Malignancy

**Bethesda**	**20-39 y**	**40-59 y**	**≥60 y**	***P***
**I, n (%)**	2 (0.6)	5 (0.9)	4(1.5)	0.563
**II, n (%)**	108(32.9)	267(50.6)	156(57.4)	<0.001
**III, n (%)**	87(26.5)	118 (22.4)	57(21)	0.221
**IV,n (%)**	7(2.1)	23 (4.4)	3(1.1)	0.021
**Malignant (V,VI), n (%)**	124(37.8)	115 (21.8)	52(19.1)	<0.001
**Confirmed malignancy, n (%) ***	150 (45.7)	153(28.9)	64 (23.5)	<0.001
**Total, n (%)**	328(29.1)	528 (46.8)	272(24.1)	
